# Combined clinical and genetic testing algorithm for cervical cancer diagnosis

**DOI:** 10.1186/s13148-016-0232-3

**Published:** 2016-06-10

**Authors:** Yu-Ligh Liou, Tao-Lan Zhang, Tian Yan, Ching-Tung Yeh, Ya-Nan Kang, Lanqin Cao, Nayiyuan Wu, Chi-Feng Chang, Huei-Jen Wang, Carolyn Yen, Tang-Yuan Chu, Yi Zhang, Yu Zhang, Honghao Zhou

**Affiliations:** Department of Clinical Pharmacology, Xiangya Hospital, Central South University, Hunan, 410078 People’s Republic of China; Department of Obstetrics and Gynecology, Xiangya Hospital, Central South University, Hunan, 410008 People’s Republic of China; Institute of Clinical Pharmacology, Hunan Key Laboratory of Pharmacogenetics, Central South University, Changsha, 410008 People’s Republic of China; Department of Obstetrics and Gynecology, Buddhist Tzu Chi General Hospital, Hualien, 97002 Taiwan; iStat Biomedical Co. Ltd., New Taipei City, 22102 Taiwan; Department of Molecular and Cell Biology, University of California at Berkeley, Berkeley, CA 94720-3200 USA; Institute of Medical Science, Tzu Chi University, Hualien, 97002 Taiwan; Center for Cervical Cancer Prevention, Department of Research Buddhist, Tzu Chi General Hospital, Hualien, 97002 Taiwan

**Keywords:** Biomarkers, Algorithm, DNA methylation, HPV16/18, Cervical cancer, *ZNF582*, *PAX1*

## Abstract

**Background:**

Opportunistic screening in hospitals is widely used to effectively reduce the incidence rate of cervical cancer in China and other developing countries. This study aimed to identify clinical risk factor algorithms that combine gynecologic examination and molecular testing (paired box gene 1 (*PAX1*) or zinc finger protein 582 (*ZNF582*) methylation or HPV16/18) results to improve diagnostic accuracy.

**Methods:**

The delta Cp of methylated *PAX1* and *ZNF582* was obtained via quantitative methylation-specific PCR in a training set (57 CIN2− and 43 cervical intraepithelial neoplasia ≥grade 3 (CIN3+) women), and the individual and combination gene sensitivities and specificities were determined. The detection accuracy of three algorithms combining gynecologic findings and genetic test results was then compared in a randomized case-control study comprising 449 women referred for colposcopic examination by gynecologists in the outpatient department of Xiangya Hospital between November 2011 and March 2013.

**Results:**

Significant association was observed between CIN3+ and methylated *PAX1* or *ZNF582* in combination with HPV16/18 (OR:15.52, 95 % CI:7.73–31.18). The sensitivities and specificities of methylated *PAX1* or *ZNF582* combined with HPV16/18 for CIN3+ women were 89.2 and 76.0 %, or 85.4 and 80.1 %, respectively. Of the three algorithms applied to cohort data and validated in the study, two indicated 100 % sensitivity in detecting cervical cancer and a low rate of referrals for colposcopy.

**Conclusions:**

These algorithms might contribute to precise and objective cervical cancer diagnostics in the outpatient departments of hospitals in countries with high mortality and low screening rates or areas with uneven resource distribution.

**Electronic supplementary material:**

The online version of this article (doi:10.1186/s13148-016-0232-3) contains supplementary material, which is available to authorized users.

## Background

Cervical cancer is the fourth most common cancer that affects women worldwide. The use of the cytological test developed by George Papanicolaou (the Pap smear) in cervical cancer screening programs has led to a reduction in the incidence of cervical cancer in developed countries [1, 2]. However, most cases of cervical cancer are still associated with absent or deficient screening [3–6]. In China, cervical cancer remains the second-leading cause of death from cancer among females aged 15 to 44 years, and nearly 58,000 new cases and 20,000 deaths were documented in 2005 alone. In certain developed cities such as Beijing and Shanghai, the incidence of cervical cancer has dropped significantly because of the wide promotion of cervical cancer prevention and opportunistic screening in hospitals. Despite these advances, prevention awareness of cervical cancer is still inadequate in most areas of China because of its large population size [7, 8].

Oncogenic high-risk human papillomavirus (hrHPV) DNA testing is currently an appealing method for the molecular diagnosis of cervical cancer, as HPV plays an essential role in cervical carcinogenesis [9–12]. In China, the application of HPV testing has increased dramatically because of its consistency and reproducibility. Thus, HPV testing has increased to the level of an “HPV-heavy-burden of testing” in certain regions, with the prevalence varying significantly among different ages and regions [13]. Consequently, progressively more HPV-positive patients are in need of cytological or colposcopic examination in hospitals, which in turn highlights the deficiencies of the examination procedure and the overloading of the performing physicians [14]. HPV-positive assay results might also cause an adverse psychosocial impact on patients. P16/Ki67 dual staining is used as a tool for CIN2 triage; however, Wentzensen et al. reported that P16/Ki67 dual staining yielded a 78.9 % positive rate for CIN2 and an 86.9 % positive rate for CIN3/CIS/cancer but also exhibited a 41.1 % positive rate for the CIN2− group [15]. Therefore, a more accurate method is required to reduce the high false-positive rate of hrHPV testing and to maintain the consistency and reproducibility of methods used in the outpatient departments of hospitals in China or other high-population countries.

One such screening possibility arises from the field of epigenetics [16]. Numerous investigations have reported that the gene-specific hypermethylation that occurs in the pre-invasive and invasive phases of cervical cancer might serve as a promising biomarker for early diagnosis [17, 18]. Several studies examining paired boxed gene 1 (*PAX1*) and zinc finger protein 582 (*ZNF582*) have reported their potential utilization as highly sensitive biomarkers for detection of cervical intraepithelial neoplasia at grade 3 or higher (CIN3+) [19–26]. The *PAX1* gene was found to be involved in the regulation of cell differentiation in head and neck cancer, and the *ZNF582* gene is a protein-coding gene involved in gene expression [27, 28].

The aim of this study was to develop and verify effective clinical risk factor algorithms to increase the accuracy of diagnosis for cervical cancer based on existing validated candidate molecular tests and the highly weighted factor of clinical examination information from the subjects. Standardized quantitative methylation-specific PCR tests of methylated PAX1 gene (*PAX1*^*m*^) and methylated ZNF582 gene (*ZNF582*^*m*^) were performed on a full spectrum of cervical scrapings, with cutoff values selected from the training set, to determine the sensitivities and specificities for CIN3+ detection in the validation set. The gynecology examination results were also considered as a prominent risk factor in the algorithms for cervical cancer prediction. We propose that these algorithms would be useful in the outpatient departments of hospitals in China or other high-population countries.

## Case presentation

### Patient recruitment and study design

In accordance with the Declaration of Helsinki, all patients provided informed consent for their participation in the clinical study, whose protocol was approved by the Institutional Review Board of the Department of Clinical Pharmacology at Xiangya Hospital, Central South University, China. The clinical trial was registered in the Chinese Clinical Trial Registry (ChiCTR-DOD-14005446). Study inclusion criteria included females who were sexually active, not pregnant, had an intact uterus, and had no history of treatment for cervical intraepithelial neoplasia (CIN) or cervical cancer. Patients with a history of cancer related to the reproductive tract or therapy for cervical lesions or HPV vaccinations, in addition to those who had a current pregnancy, were excluded.

Female patients who had abnormal Pap smears, cervical inflammation, cervical erosion, or bleeding syndrome, or those who were suspected to have cervical cancer and were referred for colposcopic examination by the gynecologist in the outpatient department of the hospital, were included in this study. Patients were invited to the colposcopic examination room of the Department of Obstetrics and Gynecology to participate in a blinded study where testers were not provided clinical data. After patients signed an informed consent form, a standardized personal interview was given by experienced assistants, a case report form was filled, and every patient received a colposcopic examination and biopsy. The case report form included the inclusion and exclusion criteria, basic personal information, historic lifestyle and sickness records, histories of gynecologic examinations and findings, cytological results, and pathological results. The cytology results were classified according to the 2001 Bethesda System (TBS 2001) [29]. Colposcopy-directed biopsies were performed to provide histological results according to standard procedures in China. Biopsy specimens were histologically classified as normal, CIN1, CIN2, CIN3, cervical carcinoma in situ (CIS), squamous cell carcinoma (SCC), or adenocarcinoma (AC), according to the international criteria. The final diagnosis was based on the results of tissue-proven pathology. To ensure the quality of the diagnosis, two expert cytologists and two pathologists independently reviewed the cytology and histology slides, respectively. Standard guidelines for the management and treatment of cervical neoplasia were followed in all subjects [30]. All patient recruitment and clinical information collection processes were periodically monitored, and good clinical practice (GCP) guidelines were followed.

In the intervention arm of the randomized-controlled trial, data from the first 100 patients were used as a training set to build a prediction model that distinguished CIN3+ subjects from controls (Additional file 1: Table S1). The validation set was composed of the subsequent 466 patients (17.6 % of the total patients in the colposcopic room), with a mean age of 42.8 years (range, 27.5–77.8 years), enrolled from November 2011 to March 2013.

Twelve patients were excluded based on the exclusion criteria, and five were excluded because of poor quality of the DNA specimens. Thus, data from 449 patients were included in the final statistical analyses. The criteria used to determine positive and negative results for the tested methylated genes were based on the delta Cp of the training set (described in further detail below).

### Specimen collection and DNA preparation

All liquid-based cytology samples were collected using CytoFast Solution (Hospitex Diagnostics SRL, Sesto Fiorentino, Italy). Residual cervical cells from cytological tests were used for HPV typing and methylation detection tests for the two genes. All specimens collected were assigned a number and delinked from patient clinical information until final data analysis. All molecular tests were performed at the Institute of Clinical Pharmacology, Hunan Key Laboratory of Pharmacogenetics, China, following good laboratory practice guidelines. The cells were centrifuged and stored in phosphate-buffered saline at −20 °C from the day of collection. Genomic DNA (gDNA) was extracted from the collected cells using the QIAamp DNA Mini Kit (Qiagen GmbH, Hilden, Germany). A BioSpec-nano spectrophotometer (Shimadzu Corporation, Tokyo, Japan) was used to quantify the amount of extracted DNA.

### DNA methylation tests

Quantitative methylation-specific PCR was performed using TaqMan-based technologies on the Lightcycler LC480 real-time PCR system (Roche Applied Sciences, Penzberg, Germany) with Cervi-P and Cervi-Z DNA detection kits (iStat Biomedical, Taipei, Taiwan). Briefly, 500 ng of gDNA was subjected to bisulfite conversion using EZ DNA Methylation-Gold Kits (Zymo Research, Irvine, CA, USA). The methylation levels of the *PAX1* and *ZNF582* genes were then determined using the qPCR kits with internal controls according to the manufacturer’s recommendations. The PCR reactions consisted of an initial incubation at 95 °C for 10 min, followed by 50 cycles of denaturation at 95 °C for 10 s and annealing and extension at 60 °C for 40 s. Fluorescence data were collected during the annealing/extension step for Cp determination.

### HPV DNA amplification and genotyping

The hrHPV-typing procedure was performed using a nested multiplex PCR assay that combined degenerate E6/E7 consensus primers and type-specific primers as previously described [31]. hrHPV type was determined after determining the size of the nested PCR amplification product.

### Algorithms for combining molecular tests and clinical gynecologic examination results

To ascertain the clinical characteristics of the subjects, items of the case report form (Table 3) including gynecologic history were answered by patients, and the gynecologic examination results were recorded by the physicians for each patient in the study. The odds ratio for each clinical characteristic was determined by univariate analysis. The significantly associated factors were then used in a multivariate logistic regression analysis to select the variables based on goodness-of-fit analysis. Multiple regression analysis revealed that a gynecologic history of vaginal bleeding, grossly normal cervix (over 90 % of the cervix had a uniform surface without warts or masses), and a finding of cervical bleeding during the gynecologic examination were associated with CIN3+ as shown in Table 3 (adjusted *P* ≤ 0.05).

First, a logistic regression analysis based on molecular tests (*PAX1*^*m*^ and/or *ZNF582*^*m*^, independently or both combined with HPV16/18) and key clinical characteristics (vaginal bleeding, grossly normal cervix, and finding of cervical bleeding) was used to discriminate between patients with CIN3+ and CIN2− cervical lesions. The methylated genes in the logistic regression formula for algorithms 1 to 3 were *PAX1*^*m*^, *ZNF582*^*m*^, and *PAX1*^*m*^ or *ZNF582*^*m*^, respectively.

The logistic regression formula was as follows: $$ \begin{array}{l}\mathrm{logistic}\ \mathrm{score} = \left[W1*\mathrm{methylated}\ \mathrm{gene}\ \mathrm{OR}\right] + \left[W2*\ HPV 16/ 18\ \mathrm{OR}\right] + \left[W3*\ \mathrm{vaginal}\ \mathrm{bleeding}\ \mathrm{OR}\right] + \left[W4*\ \mathrm{grossly}\ \mathrm{normal}\ \mathrm{cervix}\ \mathrm{OR}\right] + \\ {}\left[W5*\mathrm{contact}\ \mathrm{bleeding}\ \mathrm{OR}\right]+C\ \left(\mathrm{constant}\ \mathrm{number}\right).\end{array} $$

Second, the weight factor (W1–5; standardized regression coefficients) was calculated between the analytic results for the molecular tests and the key clinical characteristics. The weight factor value is a measure of how strongly each test influences the criterion variable (CIN3+ lesion). Each of the scores for the individual molecular tests and key clinical characteristics was entered into the logistic score as either “0” (negative) or “1” (positive). The results for the weight factor were calculated using the multivariate logistic regression.

For example:

$$ \begin{array}{l}\mathrm{Logistic}\ \mathrm{score}\ \mathrm{of}\ \mathrm{algorithm}\ 2:\ \left(2.334*ZNF 58{2}^m\mathrm{OR}\right) + \left(1.485*\mathrm{H}\mathrm{P}\mathrm{V}16/18\ \mathrm{OR}\right) + \left(0.529*\ \mathrm{vaginal}\ \mathrm{bleeding}\ \mathrm{OR}\right)\ \\ {} + \left(-0.534*\ \mathrm{grossly}\ \mathrm{normal}\ \mathrm{cervix}\ \mathrm{OR}\right) + \left(0.759*\ \mathrm{contact}\ \mathrm{bleeding}\ \mathrm{OR}\right) - 2.133.\end{array} $$

Each of the three algorithms differed in the weights and methylated genes used. Finally, the logistic score was transformed into a probability score. The probability score had a range of values from 0 to 1000, which indicated the probability of CIN3+ for each algorithm [32].

The probability score was calculated as [*e*^Logistic score^]/[1 + *e*^Logistic score^] * 1000.

### Statistical analysis

The cutoff values for each methylated gene were generated from the first 100 subjects, including 43 with CIN3+ results and 57 with CIN2− results. A cross-validated receiver operating characteristic (ROC) curve was generated, and the area under the ROC curve (AUC) was calculated for each detection method for CIN3+ lesions. The optimal cutoff value, i.e., the delta crossing point (ΔCp), of each methylated gene was generated using the Youden index. The positive cutoff values for *PAX1*^*m*^ and *ZNF582*^*m*^ were determined as ΔCp ≤ 9 and ΔCp ≤ 11, respectively, from the first 100 subjects. SPSS software (version 16.0, Chicago, IL, USA) was used for all statistical analyses. Chi-squared and Fisher’s exact tests were used to analyze the status of the methylated genes and HPV genotype in different combinations. Fisher’s exact test is considered more accurate than the chi-squared test when the sample size is smaller than five. The sensitivity, specificity, and odds ratio (OR) with a 95 % confidence interval (CI) for lesions of grade CIN3 or worse were calculated. All differences were considered two-sided and statistically significant at *P* < 0.05. The algorithms were based on the multivariate logistic regression model.

### Discussion and evaluation

Several previous studies have indicated that the analysis of *PAX1*^*m*^ and *ZNF582*^*m*^ in cervical cell scrapings and tissues holds great promise for detecting high-grade CIN lesions and cervical cancer [19, 22]. However, these studies were conducted on selected populations such as within an outpatient referral case control study or following triage using cytology or hrHPV, wherein cytology was examined solely in LSIL patients. These study designs hampered the proper comparison of molecular test performance with cytology results, as patients with normal or partially abnormal results did not receive follow-up exams. The strengths of the present study lie in its incorporation of delinked random case-control study methods incorporating all histological results, including those of the normal group. This enabled the comparison of the results obtained with methylation markers with those of cytology or HPV genotyping for cervical cancer screening.

To our knowledge, this study is the first report to validate an opportunistic cervical cancer screening method that utilizes gynecologic examination findings, gynecologic history, and genetic biomarkers in combination to increase the accuracy of diagnoses under hospital outpatient conditions. In addition, we consider that this study also provides the first suggestion that that the majority of cervical cancer diagnoses in China or other developing countries should be obtained through opportunistic screening. Our results demonstrated that of the two genes used in the study, methylated *ZNF582*, with 76.6 % sensitivity and 86.94 % specificity for detecting cervical cancer, is a more promising biomarker. *ZNF582*^*m*^ has been reported to function well in the triage of patients with equivocal liquid-based cytology results [19, 25]. The second tested biomarker, *PAX1*^m^, has also been reported as a useful biomarker for cervical cancer in the screening and triage of cytology and for the detection cervical adenocarcinoma. The results of this meta-analysis support the utility of *PAX1*^m^ as an auxiliary biomarker in cervical cancer screening [33], as algorithm 1 demonstrated 89.87 % sensitivity and 75.95 % specificity for *PAX1*^*m*^ in combination with HPV16/18 and cytology testing, which is higher than the values obtained by testing any of these factors alone.

Both algorithms 2 and 3 showed a 100 % detection rate in the cancer groups. We therefore recommend a new cervical cancer patient management strategy consisting of both algorithms 2 and 3 for use in opportunistic screening in hospitals. Patients who exhibit positive test results from algorithm 2 or 3 should then undergo a colposcopy examination or a biopsy. Upon obtaining negative results, patients should return for follow-up examinations at 6 months or 1 year. As a cancer management strategy, algorithm 2 might reduce the number of hrHPV-positive patients referred for colposcopy by 38.5 %, whereas algorithm 3 might reduce the referral number by 27.56 %; however, algorithm 3 also had an 83.33 % positive rate for the histologic CIN3 category, which is 13.89 % higher than that obtained with algorithm 2.

In this study, the sensitivity and specificity of hrHPV testing and Pap smear tests applied individually for detecting CIN3+ were 98.1 and 46.1 %, and 69.0 and 90.7 %, respectively. However, in China, cytology and hrHPV diagnosis are time-consuming and impractical because of inaccurate results. Testing is performed in many hospitals that do not have sufficient medical resources to perform colposcopy and additional examinations, thus impeding proper diagnosis. Furthermore, colposcopy is invasive and causes anxiety in many patients. In comparison, the three proposed algorithms combine *PAX1*^*m*^ and/or *ZNF582*^*m*^ with HPV16/18 testing and take into account gynecologic history/examination findings to enhance accuracy. These algorithms could improve the positive detection rate of CIN3+ lesions, with clinical observation and gene testing both proposed as follow-up measures. The use of these algorithms might thus greatly reduce the referral rate of hrHPV-positive patients and increase the accuracy of cytology in countries with limited resources for colposcopy.

The present study has some potential limitations. For example, the subjects who were recruited were seen following referral for colposcopy examination and consisted of patients who had abnormal Pap smear results, inflammation syndrome, cervical erosion, bleeding syndrome, or suspected cervical cancer. In our cohort, >90 % of the patients had inflammation syndrome with positive hrHPV findings, which is not representative of the general population. In addition, many ASC-US patient samples were collected in the colposcopy room because most patients with obvious cervical cancer underwent biopsy immediately following abnormal Pap smear test results and clinical observation in the outpatient department. Other limitations include a small sample size and a lack of extensive and long-term follow-up information.

### Patient clinicopathological characteristics

Cohort demographic characteristics, clinical information, and *PAX1*^*m*^ and *ZNF582*^*m*^ testing results are shown in Table 1. The cytology results of the cohort indicated that 99 were normal, 208 had atypical squamous cells of undetermined significance (ASC-US), 6 exhibited low-grade squamous intraepithelial lesion (LSIL), and 136 had atypical squamous cells for which HSIL/atypical glandular cell of undetermined significance/high-grade squamous intraepithelial lesion+ (ASC-H/AGC/HSIL+) status could not be excluded. In total, colposcopy and biopsy revealed normal histology in 218 (48.55 %) patients, CIN1 in 30 (6.68 %) patients, CIN2 in 43 (9.58 %) patients, CIN3 in 72 (16.04 %) patients, CIS in 15 patients (3.34 %), and SCC/AC in 71 (15.81 %) patients. The positive rates of *PAX1*^*m*^ and *ZNF582*^*m*^ in the CIN3+ group were 69.6 and 77.6 %, respectively.

**Table 1:** Population and test characteristics by histologic category

		Histological results						Total
	Cutoff	Normal	CIN1	CIN2	CIN3	CIS	SCC/AC	
Number of subjects								
*N* (%)		218 (48.55)	30 (6.68)	43 (9.58)	72 (16.04)	15 (3.34)	71 (15.81)	449 (100)
Age (years)								
Mean ± SD (range)		41.2 ± 10.6 (21.5 to 77.8)	47.0 ± 9.6 (29.0 to 66.2)	39.2 ± 11.1 (21.8 to 64.6)	37.3 ± 5.1 (23.7 to 63.0)	45.1 ± 8.7 (28.3 to 65.7)	42.9 ± 4.5 (32.5 to 74.3)	42.8 ± 10.3 (21.5 to 77.8)
Cytology results								
Normal (%)		90 (90.91)	4 (4.04)	2 (2.02)	0 (0.00)	1 (1.01)	2 (2.02)	99 (100)
ASC-US (%)		112 (53.85)	21 (10.10)	32 (15.38)	24 (11.54)	5 (2.40)	14 (6.73)	208 (100)
LSIL (%)		1 (16.67)	2 (33.33)	0 (0.00)	3 (50.00)	0 (0.00)	0 (0.00)	6 (100)
ASC-H/AGC/HSIL+ (%)		15 (11.03)	3 (2.21)	9 (6.62)	45 (33.09)	9 (6.62)	55 (40.44)	136 (100)
Detection modality or test used								
hrHPV (%)		100 (45.87)	18 (60.00)	39 (90.70)	71 (98.61)	15 (100)	69 (97.18)	312 (69.49)
HPV16/18 (%)		19 (8.72)	5 (16.67)	12 (27.91)	36 (50.00)	11 (73.33)	56 (78.87)	139 (30.96)
*PAX1* ^*m*^ (%)	ΔCp ≦ 9.0	34 (15.60)	4 (13.33)	15 (34.88)	47 (65.28)	11 (73.33)	52 (73.24)	163 (36.30)
*ZNF582* ^*m*^ (%)	ΔCp ≦ 11.0	22 (10.09)	6 (20.00)	10 (23.26)	48 (66.67)	12 (80.00)	61 (85.92)	159 (35.41)

### Analytic sensitivity and specificity of the methylation analysis combined with HPV genotyping in the validation set

Table 2 shows the results of the sensitivity, specificity, and accuracy for different molecular tests and their combinations for detection of CIN3+ lesions. The AUCs of HPV16/18, *PAX1*^*m*^, and *ZNF582*^*m*^ in the validation set were 76.4, 75.5, and 81.8 %, respectively. The sensitivity and specificity of HPV16/18 for detecting CIN3+ lesions were 65.19 and 87.63 %, respectively, whereas *PAX1*^*m*^ showed 69.6 % sensitivity (95 % CI 62.1–76.3) and 81.8 % specificity (95 % CI 77.0–85.8), and *ZNF582*^*m*^ exhibited 76.6 % sensitivity (95 % CI 69.4–83.0) and 86.9 % specificity (95 % CI 82.6–90.3). We therefore analyzed *PAX1*^*m*^ and *ZNF582*^*m*^ as reliable molecular tests in comparison to Pap smear results.

**Table 2:** Performance of methylated genes and HPV tests for CIN3+ detection

Tests	Sensitivity (%) (95 % CI)	Specificity (%) (95 % CI)	AUC (%) (95 % CI)	Odds ratio (95 % CI)	*P* value
*PAX1* ^*m*^	69.62 (62.05–76.26)	81.79 (76.95–85.80)	75.5 (70.8–80.6)	10.29 (6.55–16.16)	<0.001
*ZNF582* ^*m*^	76.58 (69.40–82.51)	86.94 (82.58–90.34)	81.8 (77.3–86.2)	21.77 (13.18–35.96)	<0.001
PAP	68.99 (61.40–75.68)	90.72 (86.84–93.54)	79.9 (75.1–84.6)	21.75 (12.93–36.59)	<0.001
hrHPV	98.10 (94.57–99.35)	46.05 (40.41–51.79)	72.1 (67.5–76.7)	44.10 (13.75–141.45)	<0.001*
HPV-16/18	65.19 (57.48–72.18)	87.63 (83.35–90.93)	76.4 (71.4–81.4)	13.27 (8.22–21.40)	<0.001
*PAX1* ^*m*^ or hrHPV	98.73 (95.50–99.65)	42.61 (37.06–48.35)	70.7 (66.0–75.3)	57.92 (14.09–238.17)	<0.001*
*PAX1* ^*m*^ or HPV-16/18	89.24 (83.45–93.17)	75.95 (70.72–80.50)	82.6 (78.5–86.6)	26.19 (14.80–46.33)	<0.001
*PAX1* ^*m*^ or *ZNF582* ^*m*^	85.44 (79.10–90.10)	76.98 (71.81–81.44)	81.2 (76.9–85.5)	19.62 (11.67–32.99)	<0.001
*ZNF582* ^*m*^ or hrHPV	98.73 (95.50–99.65)	45.02 (39.40–50.76)	71.9 (67.3–76.5)	63.86 (15.53–262.56)	<0.001*
*ZNF582* ^*m*^ or HPV-16/18	85.44 (79.10–90.10)	81.10 (76.21–85.18)	83.3 (79.2–87.4)	25.19 (14.82–42.82)	<0.001

*P* value determined by chi-squared test and *Fisher’s exact test; odds ratio for CIN3+

*CI* confidence interval, *HPV* human papillomavirus, *hrHPV* high-risk human papillomavirus, *Gene* ^*m*^ methylated gene

In the combined parallel testing of gene methylation with HPV genotyping, *ZNF582*^*m*^ and *PAX1*^*m*^ combined with HPV16/18 results exhibited an AUC >82 % for detection of CIN3+. *ZNF582*^*m*^ combined with HPV16/18 showed the best combination of high sensitivity (85.4 %) and high specificity (81.1 %), whereas *PAX1*^*m*^ combined with HPV16/18 had the highest sensitivity of 89.2 % and a specificity of 76.0 %. The combination of both methylated genes, *ZNF582*^*m*^ and *PAX1*^*m*^, exhibited 85.4 % sensitivity and 77.0 % specificity.

### Algorithms combining gynecologic examination findings and genetic test results

The crude ORs and corresponding 95 % CIs for prediction of CIN3+ lesions in the study cohort were calculated. Univariate analyses revealed that age, number of pregnancies, contraceptive measures such as condom use, gynecologic history, and certain gynecologic examination findings were significantly (*P* < 0.05) associated with CIN3+, as shown in Table 3. Patients who were over 50 years old (OR:2.97, 95 % CI:1.13–7.8) and those from 30 to 50 years of age (OR:2.13, 95 % CI:0.68–6.69) were found to have a higher risk of CIN3+ lesions compared to patients under 30 years of age. Patients who had given birth over three times or had a cervical mass finding on tumor examination were still considered to be at risk for cervical cancer. In the latter analysis, patients who had a gynecologic history of vaginal bleeding and examination findings of contact bleeding had an approximately 3.77- to 2.95-fold higher risk of CIN3+ lesions compared to those without these characteristics. In contrast, the gynecologic examination finding of a grossly normal cervix was negatively associated with CIN3+ lesions; furthermore, such patients exhibited a 50 % lower risk of CIN3+ lesions compared to those with morphology changes.

**Table 3:** Crude and adjust odds ratios (ORs) and corresponding 95 % confidence intervals (CIs) for predictors of CIN3+ in cervical cancer

	Odds ratio (95 % CI)				
Variable	Number	Crude	*P* value	Adjusted	*P* value
Age group					
<30	52	Reference		Reference	
30–50	319	3.93 (1.72–9.00)	0.001	2.97 (1.13–7.80)	0.027
>50	78	4.02 (1.61–10.06)	0.003	2.13 (0.68–6.69)	0.194
No. of pregnancies					
0	24	Reference			
1~3	232	2.68 (0.89–8.11)	0.081		
>3	193	3.04 (1.00–9.25)	0.050		
No. of births					
0	46	Reference		Reference	
1~3	363	2.24 (1.05–4.79)	0.038	1.06 (0.43–2.63)	0.895
>3	40	4.54 (1.75–11.83)	0.002	1.71 (0.53–5.56)	0.370
Intrauterine device (IUD) usage					
No	215	Reference			
Yes	234	1.41 (0.95–2.08)	0.087		
Condom usage					
No	336	Reference		Reference	
Yes	113	0.59 (0.37–0.94)	0.027	0.78 (0.46–1.31)	0.349
Oral contraception usage					
No	391	Reference			
Yes	58	0.81 (0.45–1.46)	0.478		
Tubal ligation					
No	360	Reference			
Yes	89	0.98 (0.60–1.60)	0.937		
Gynecology history: cervical contact bleeding					
No	329	Reference		Reference	
Yes	120	1.61 (1.05–2.47)	0.030	1.11 (0.68–1.80)	0.673
Gynecology history: vaginal bleeding					
No	385	Reference		Reference	
Yes	64	3.77 (2.18–6.54)	<0.001	2.95 (1.62–5.39)	<0.001
Gynecology history: abnormal vaginal discharge					
No	439	Reference			
Yes	10	1.87 (0.53–6.56)	0.329		
Gynecologic examination: grossly normal cervix					
No	323	Reference		Reference	
Yes	126	0.40 (0.25–0.65)	<0.001	0.51 (0.30–0.85)	0.011
Gynecologic examination : cervical erosion					
No	196	Reference			
Yes	253	1.00 (0.68–1.48)	0.995		
Gynecologic examination: cervical mass					
No	349	Reference		Reference	
Yes	100	2.01 (1.28–3.15)	0.003	1.37 (0.83–2.25)	0.220
Gynecologic examination: ulcer					
No	446	Reference			
Yes	3	3.72 (0.33–41.33)	0.285		
Gynecologic examination: contact bleeding					
No	403	Reference		Reference	
Yes	46	2.95 (1.58–5.50)	0.001	1.94 (0.99–3.83)	0.055

**Fig. 1:**
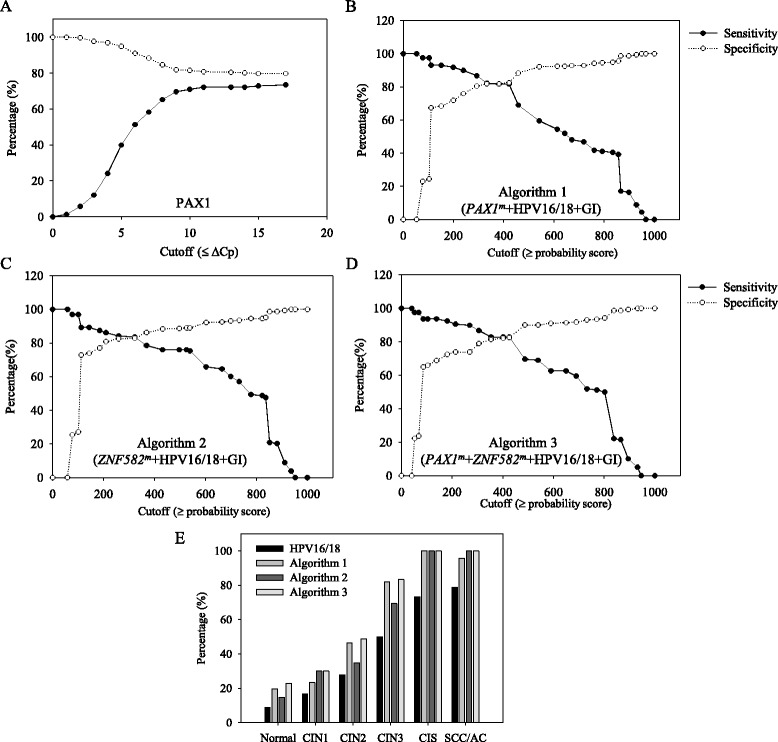
Representative values plotted versus cutoff values in cervical cancer. **a**–**d** Illustrate the sensitivity and specificity of tests (*PAX1* ^*m*^ and algorithms) at different cutoff values for detecting CIN3+ lesions. **e** Bar chart showing the positivity rate for algorithms in each histologic category when 220 was used as a cutoff value. *GI* gynecologic information

Figure 1a shows the sensitivity and specificity of the *PAX1*^*m*^ gene only and the positive results determined at the ΔCp ≤ cutoff value. Detection of the *PAX1*^*m*^ gene only had a limited sensitivity of approximately 80 %. Figure 1b–d shows the range of cutoff values (probability score) of 0 to 1000 generated for algorithm 1 to 3, and the sensitivity and specificity of each algorithm were plotted with the different cutoff values (probability score). Finally, the optimal probability score was determined to be 220 in algorithms 1 to 3 by Youden’s J statistic.

The bar chart in Fig. 1e shows the proportion of HPV 16/18 and the detection rate for the other three algorithms with each histologic category. The positive detection rates for CIN3+ of the three algorithms were higher than those for HPV16/18 alone. All algorithms demonstrated over 86 % sensitivity and 72 % sensitivity for the detection of CIN3+ lesions (Table 4). Algorithm 1, which showed 89.87 % sensitivity and 75.95 % specificity, was 24.7 % more sensitive and 11.7 % less specific than HPV16/18 testing alone for detecting CIN3+ lesions, whereas algorithms 2 and 3 were 20.9 and 27.22 % more sensitive and 6.9 and 15.12 % less specific than HPV16/18, respectively.

**Table 4:** Sensitivity, specificity, and odds ratios of the three clinical risk factor algorithms for CIN3+ or CIS/SCC/AC detection

Target genes	Cutoff	Sensitivity (%) (95 % CI)	Specificity (%) (95 % CI)	AUC (%) (95 % CI)	Odds ratio (95 % CI)	*P* value
CIN3+ lesion						
Algorithm 1	220	89.87 (84.18–93.67)	75.95 (70.72–80.50)	82.9 (78.9–86.9)	28.02 (15.65–50.17)	<0.001
Algorithm 2	220	86.08 (79.82–90.62)	80.76 (75.84–84.87)	83.4 (79.3–87.5)	25.94 (15.17–44.36)	<0.001
Algorithm 3	220	92.41 (87.19–95.60)	72.51 (67.11–77.32)	82.5 (78.5–86.4)	32.09 (16.88–61.00)	<0.001
CIS/SCC/AC						
Algorithm 1	220	96.51 (90.24–98.81)	64.46 (59.41–69.21)	78.8 (74.2–83.5)	50.19 (15.55–161.98)	<0.001*
Algorithm 2	220	100.0 (94.87–100.0)	70.80 (65.92–75.24)	84.0 (80.4–87.6)	–	–
Algorithm 3	220	100.0 (94.87–100.0)	61.43 (56.33–66.30)	79.5 (75.3–83.7)	–	–

*P* value determined by chi-squared test and *Fisher’s exact test; odds ratio for CIN3+ or CIS/SCC/AC

*CI* confidence interval, *Gene* ^*m*^ methylated gene

For cancer detection, HPV16/18 had 77.90 % sensitivity. Algorithm 1 showed 95.77 % sensitivity, and algorithms 2 and 3 both exhibited 100 % sensitivity. Algorithm 2 showed a 23.42 % increase in sensitivity over *ZNF582* testing alone for the histologic cancer (CIS/SCC/AC) category but showed only a 4.58 % increase in sensitivity for detecting patients within the normal histologic category. Algorithm 2 also demonstrated a 22.10 % increase in sensitivity for the histologic cancer (CIS/SCC/AC) category over HPV16/18 tests alone. Comparison of the hrHPV-positive rates obtained with the three algorithms with that of the CIN2− group indicated a decrease in the positivity rate obtained using algorithms 1–3 to 29.9, 34.71, and 26.46 %, with a cancer detection rate of 95.77, 100, and 100 %, respectively.

Because of the large population, screening programs for cervical cancer are difficult to implement in China. However, the incidence of cervical cancer has decreased to a greater degree in urban areas than in rural areas because of the implementation of opportunistic screening in hospitals. Pap smear testing has been the major tool used for opportunistic screening in China over the past several decades. A survey of 202,231 patients in a retrospective opportunistic-screening study of 12 of the 3A hospitals in 2005 demonstrated that liquid-based cytology remained the major screening method in China [34], whereas hrHPV co-testing with cytology was utilized for approximately 11.7 % of patients in the study. Accordingly, physicians face problems in delivering accurate diagnoses such as a low positive cytologic detection rate and a high false-positive rate of hrHPV detection, which result in insufficient treatment or over-treatment, respectively. To address these concerns, we suggest that combining gene testing with gross clinical history/examination findings in the outpatient departments of hospitals would provide a first step toward reducing the incidence of cervical cancer in China.

## Conclusions

Algorithms that include molecular tests (methylated *PAX1*, *ZNF582*, and HPV16/18) in combination with clinical examination findings provide an effective method to increase the accuracy of diagnosis for cervical cancer. In this study, we established and validated algorithms that could be used as an objective screening method in the outpatient departments of hospitals to reduce the numbers of patients with cervical cancer. These algorithms might lead to the establishment of accurate, objective, non-morphological, and molecular-based test systems for cervical diagnosis in developing countries or countries where resources are not evenly distributed.

## Abbreviations

AC, adenocarcinoma; AGC, atypical glandular cell of undetermined significance; ASC-H, atypical squamous cells cannot exclude high-grade squamous intraepithelial lesions; ASC-US, atypical squamous cells of undetermined significance; AUC, area under the curve; CI, confidence interval; CIN, cervical intraepithelial neoplasia; CIS, carcinoma in situ; GCP, good clinical practice; GLP, good laboratory practice; hrHPV, high-risk human papillomavirus; HSIL, high-grade squamous intraepithelial lesion; ORs, odds ratios; Pap smear, Papanicolaou smear; *PAX1*, paired box gene 1; *PAX1*^*m*^, methylated *PAX1* gene; ROC, receiver operating characteristic; SCC, squamous cell carcinoma; *ZNF582*, zinc finger protein 582; *ZNF582*^*m*^, methylated *ZNF582* gene; PBS, Phosphate-buffered saline; LBC, liquid-based cytological test 

## Additional files

Additional file 1: Table S1. Population and test characteristics by histologic category in training set. (PPTX 47 kb)
