# Supplementary material for: Combined clinical and genetic testing algorithm for cervical cancer diagnosis
Source: Clin Epigenetics. 2016 Jun 10;8:66. doi: 10.1186/s13148-016-0232-3 (PMC4902988; doi:10.1186/s13148-016-0232-3)
Supplement: Additional file 1: Table S1. — Population and test characteristics by histologic category in training set. (PPTX 47 kb) [file 13148_2016_232_MOESM1_ESM.pptx]

## Slide 1
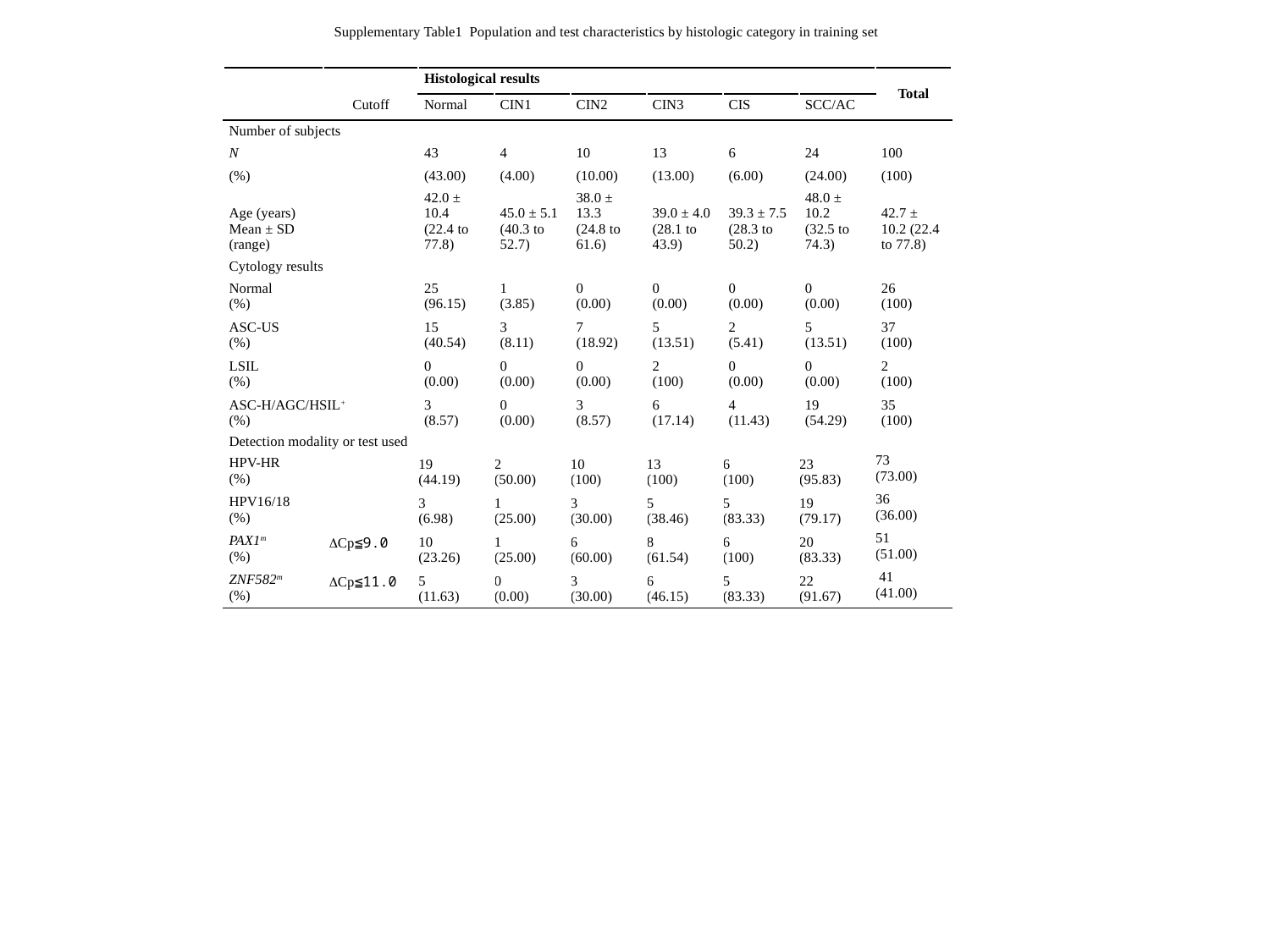

Supplementary Table1 Population and test characteristics by histologic category in training set
| | | Histological results | | | | | | Total |
| --- | --- | --- | --- | --- | --- | --- | --- | --- |
| | Cutoff | Normal | CIN1 | CIN2 | CIN3 | CIS | SCC/AC | |
| Number of subjects | | | | | | | | |
| N | | 43 | 4 | 10 | 13 | 6 | 24 | 100 |
| (%) | | (43.00) | (4.00) | (10.00) | (13.00) | (6.00) | (24.00) | (100) |
| Age (years) Mean ± SD (range) | | 42.0 ± 10.4 (22.4 to 77.8) | 45.0 ± 5.1 (40.3 to 52.7) | 38.0 ± 13.3 (24.8 to 61.6) | 39.0 ± 4.0 (28.1 to 43.9) | 39.3 ± 7.5 (28.3 to 50.2) | 48.0 ± 10.2 (32.5 to 74.3) | 42.7 ± 10.2 (22.4 to 77.8) |
| Cytology results | | | | | | | | |
| Normal (%) | | 25 (96.15) | 1 (3.85) | 0 (0.00) | 0 (0.00) | 0 (0.00) | 0 (0.00) | 26 (100) |
| ASC-US (%) | | 15 (40.54) | 3 (8.11) | 7 (18.92) | 5 (13.51) | 2 (5.41) | 5 (13.51) | 37 (100) |
| LSIL (%) | | 0 (0.00) | 0 (0.00) | 0 (0.00) | 2 (100) | 0 (0.00) | 0 (0.00) | 2 (100) |
| ASC-H/AGC/HSIL+ (%) | | 3 (8.57) | 0 (0.00) | 3 (8.57) | 6 (17.14) | 4 (11.43) | 19 (54.29) | 35 (100) |
| Detection modality or test used | | | | | | | | |
| HPV-HR (%) | | 19 (44.19) | 2 (50.00) | 10 (100) | 13 (100) | 6 (100) | 23 (95.83) | 73 (73.00) |
| HPV16/18 (%) | | 3 (6.98) | 1 (25.00) | 3 (30.00) | 5 (38.46) | 5 (83.33) | 19 (79.17) | 36 (36.00) |
| PAX1m (%) | DCp≦9.0 | 10 (23.26) | 1 (25.00) | 6 (60.00) | 8 (61.54) | 6 (100) | 20 (83.33) | 51 (51.00) |
| ZNF582m (%) | DCp≦11.0 | 5 (11.63) | 0 (0.00) | 3 (30.00) | 6 (46.15) | 5 (83.33) | 22 (91.67) | 41 (41.00) |
